# Effects of high-frequency biphasic shocks on ventricular vulnerability and defibrillation outcomes through synchronized virtual electrode responses

**DOI:** 10.1371/journal.pone.0232529

**Published:** 2020-05-01

**Authors:** Yu-An Chiou, Li-Kuan Cheng, Shien-Fong Lin

**Affiliations:** 1 Department of Electrical and Computer Engineering, College of Electrical and Computer Engineering, National Chiao Tung University, Hsinchu, Taiwan; 2 Institute of Biomedical Engineering, College of Electrical and Computer Engineering, National Chiao Tung University, Hsinchu, Taiwan; University of Minnesota, UNITED STATES

## Abstract

Electrical defibrillation is a well-established treatment for cardiac dysrhythmias. Studies have suggested that shock-induced spatial sawtooth patterns and virtual electrodes are responsible for defibrillation efficacy. We hypothesize that high-frequency shocks enhance defibrillation efficacy by generating temporal sawtooth patterns and using rapid virtual electrodes synchronized with shock frequency. High-speed optical mapping was performed on isolated rat hearts at 2000 frames/s. Two defibrillation electrodes were placed on opposite sides of the ventricles. An S1-S2 pacing protocol was used to induce ventricular tachyarrhythmia (VTA). High-frequency shocks of equal energy but varying frequencies of 125–1000 Hz were used to evaluate VTA vulnerability and defibrillation success rate. The 1000-Hz shock had the highest VTA induction rate in the shorter S1-S2 intervals (50 and 100 ms) and the highest VTA defibrillation rate (70%) among all frequencies. Temporal sawtooth patterns and synchronous shock-induced virtual electrode responses could be observed with frequencies of up to 1000 Hz. The improved defibrillation outcome with high-frequency shocks suggests a lower energy requirement than that of low-frequency shocks for successful ventricular defibrillation.

## Introduction

Ventricular tachyarrhythmia (VTA), which includes ventricular tachycardia and ventricular fibrillation, is a major cause of sudden cardiac death [1]. Regarding the progress of cardiac research in recent decades, electric defibrillation has been demonstrated to be the most effective life-saving tool for terminating VTA. However, the mechanism of defibrillation remains insufficiently understood because the shocks obscure conventional electrical instrumentation recordings during defibrillation. Over the past two decades, the application of optical mapping has enabled direct observation of tissue responses to electric shocks during defibrillation, thus providing new insights into complicated VTA activation patterns and defibrillation mechanisms [2–5].

Two major theories have been developed at the cell and tissue levels. The first theory at the cell level is the sawtooth hypothesis, which posits that a chain of cells interconnected by high-resistance gap junctions can generate a sawtooth pattern during the shock [6–9]. The high resistance of the gap junction on a cell membrane forces the current to flow preferentially through the cell membrane. After the current enters the cell, the nearest cathode at the end of the cell depolarizes, the anode hyperpolarizes, and a sawtooth pattern can be established by tracing the current changes in the transmembrane potential [10, 11]. Therefore, the electrical energy can propagate into tissue deeper than the superficial layers when the shock energy follows the cell bundles in accordance with the sawtooth mechanism. On the basis of the sawtooth model, if an electric field is sufficiently strong, the transmembrane potential in the depolarized areas of all cells reaches the activation threshold and initiates local excitation, which spreads to the hyperpolarized areas, and the entire cell chain is activated almost simultaneously [12]. In this study, the temporal sawtooth pattern was observed at the tissue level during high-frequency defibrillation. This phenomenon may support the defibrillation propagation mechanism and improve defibrillation efficacy.

The second major theory at the tissue level is the virtual electrode hypothesis, which was established on the basis of the bidomain model [13] and optical mapping experiments [14]. Virtual electrode theory suggests four modes of cardiac tissue excitation during defibrillation. Wikswo et al. [15] described the main illustration and details of this theory. These propagation models can be observed in experiments by using optical mapping. In optical mapping experiments under real cathode stimulation, the cardiac tissue near the electrodes depolarizes, which generates two virtual anodes and causes lateral myocardium hyperpolarization. This phenomenon explains how remote tissue not directly under the shocking electrodes can have transmembrane potential responses from the shock [12, 16, 17]. The virtual electrode hypothesis is more applicable to biphasic shocks than to monophasic shocks possibly because of the immediate phase reversal of tissue responses during the shock [15, 18]. Therefore, the multiphase reversal may further enhance defibrillation efficacy [12]. However, the virtual electrode response of multiphase defibrillation waveforms has not been observed in studies. The multiphase virtual electrode pattern was validated through experimentation in the present study and could enhance defibrillation efficacy.

Relevant studies have focused mainly on defibrillation voltage, energy, and duration [19–21]. By contrast, the present study investigated tissue response to different frequencies of defibrillation shocks. By creating a defibrillation system with adjust frequency, we applied defibrillation shocks with different frequencies (125–1000 Hz) in isolated rat hearts and tested the hypothesis that the temporal sawtooth pattern induced by high-frequency shocks helps maintain tissue polarization and refractoriness. Furthermore, the generation of rapid virtual electrode reversal synchronized with the shock frequency at remote regions may improve defibrillation efficacy. In this study, hearts were more vulnerable to high-frequency waveforms, which have greater defibrillation efficacy than low-frequency waveforms. The temporal sawtooth and spatial virtual electrode phenomena experimentally observed using optical mapping could be the underlying mechanisms of improving defibrillation outcomes.

## Material and methods

### Heart preparation

**Fig 1:**
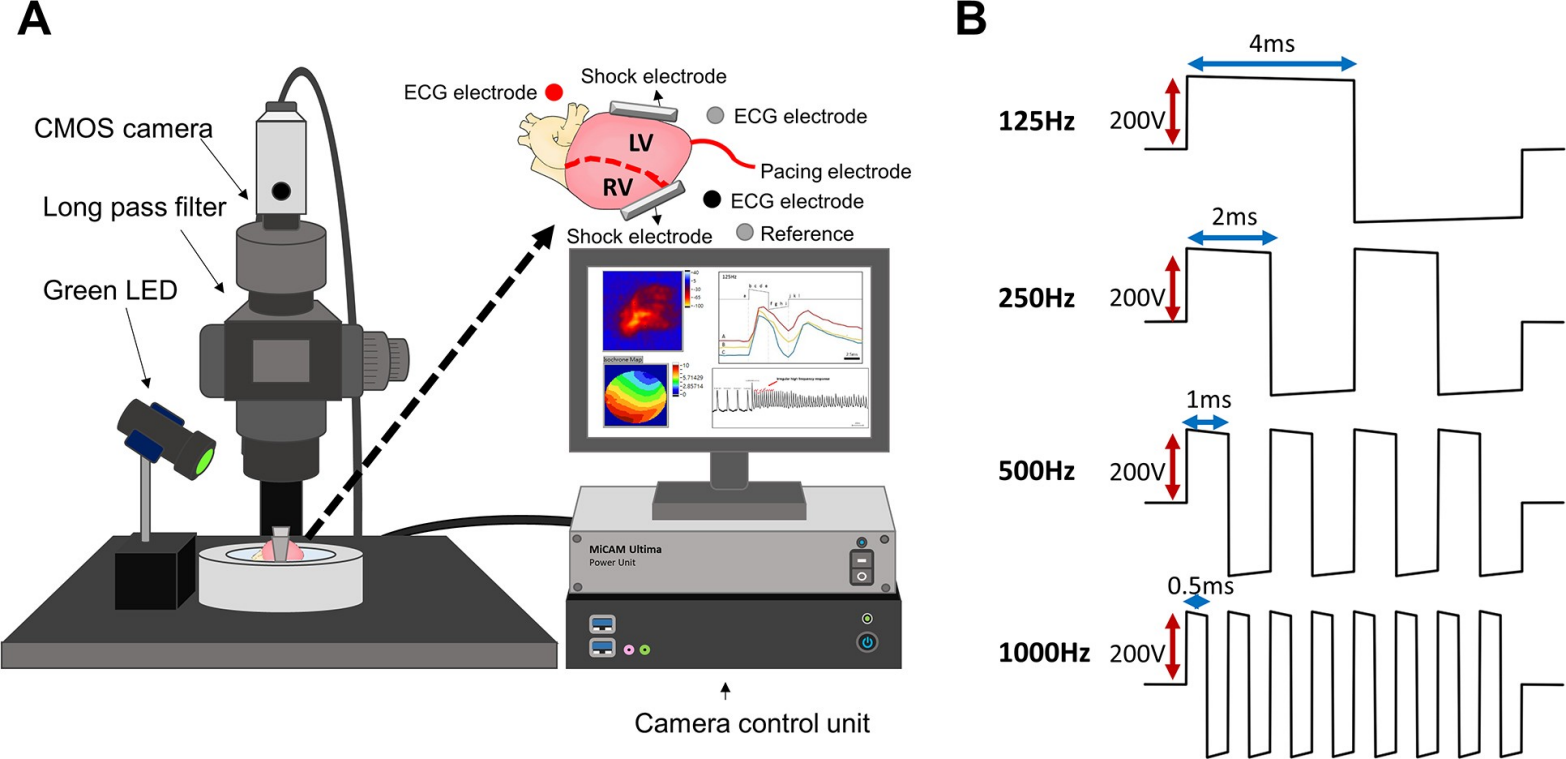
Optical mapping setting and typical waveform illustrations. (A) High-speed optical mapping setup for fluorescence imaging of isolated hearts. The inset figure displays the shock electrode placement on the lateral sides of the ventricles. (B) Four shock waveforms with different frequencies that were used as the S2s in the S1-S2 stimulation protocol.

The experimental protocol was approved by the National Chiao Tung University Institution Animal Care and Use Committee. The animal experiment was approved by the Laboratory Animal Center of National Chiao Tung University (NCTU-IACUC-107025). Female Sprague Dawley rats (n = 6) aged 8 to 10 weeks and weighing 200 to 300 g were obtained from BioLASCO Taiwan Yi-Lan Breeding Center. Rats were housed in specific pathogen-free conditions in the animal care facility at the Laboratory Animal Center of National Chiao Tung University in accordance with institutional guidelines. Animals received food and water ad libitum and were acclimatized to standard laboratory conditions (25°C ± 3°C, 55% ± 5% humidity, and a 12-h light/dark cycle). Rats were first anesthetized using isoflurane and then injected with heparin for blood anticoagulation and urethane for deeper anesthetization. Hearts were rapidly excised and subject to retrograde perfusion via the aorta with Tyrode’s solution. The Tyrode’s buffer solution composition was (in mM) 125 NaCl, 4.5 KCl, 0.5 MgCl_2_, 0.54 CaCl_2_, 1.2 NaH_2_PO_4_, 24 NaHCO_3_, and 5.5 glucose plus 50 mg/L albumin, and the pH value was adjusted to 7.4 [22]. Hearts were perfused using Tyrode’s solution at 37°C for 10 min before being stained with voltage-sensitive dyes (di-4-ANEPPS, 5 μg/mL) for fluorescence imaging and blebbistatin (10–15 μM) to inhibit heart contraction [23]. Blebbistatin is a commonly used uncoupling agent for reducing heart contraction artifacts during optical mapping. This agent is effective in inhibiting contraction while minimally affecting the optical mapping results for rodent hearts [24, 25]. A custom-designed high-power light-emitting diode with a center wavelength of 505 nm was used to excite the stained heart. The optical mapping setting is illustrated in Fig 1A. The green LED was used to induce fluorescence, which was captured through a 600-nm long-pass filter by using a high-speed camera system (MiCAM ULTIMA, SciMedia, CA, USA). The camera had an acquisition frame rate of 2000 frames/s with a frame interval of 0.5 ms. The image resolution was 100 × 100 pixels with a spatial resolution of 0.35 × 0.35 mm^2^/pixel. The images were continually acquired for 2 seconds before applying the shock waveforms. The action potential amplitudes measured using optical recording were represented by the relative change in fluorescence intensity [26]. The average intensity of the acquired image sequence was calculated to obtain a mean intensity frame, and the relative change in fluorescence intensity in consecutive frames from the “mean” was regarded as the optical potential of tissue responses.

### Experimental design

The experimental setup for rat hearts is presented in Fig 1A. The isolated heart was placed in a perfusion chamber with the anterior facing the camera objective lens. Each rat heart was electrically paced using a Teflon-coated stainless steel wire (0.3 mm in diameter) inserted into the apex as the cathode and another wire in the tissue bath as the anode. Defibrillation shocks were delivered through two stainless steel plates (5 × 10 mm^2^ in size) placed next to the left ventricle, and the cathode was next to the right ventricle. Electrocardiography (ECG) was monitored using three record electrodes placed in the heart chamber near the periphery of the heart. A custom-designed high-frequency defibrillator generated multiphasic truncated exponential decaying waveforms [27]. As illustrated in Fig 1B, the defibrillator was designed to provide four different frequencies of biphasic waveform shocks of approximately 200 V to the isolated rat heart; this design was similar to the voltage settings in an isolated rabbit heart defibrillation experiment [28]. The power of each shock was 0.45–0.51 J, which was similar to that of previous studies [29]. The output waveforms exhibited four different temporal patterns, including the conventional biphasic 125 Hz as well as 250, 500, and 1000 Hz. The four frequencies are multiples of 125 Hz with a total output duration of 8 ms. Therefore, the duration of each individual phase in the biphasic waveforms was calculated to be 4 ms at 125 Hz, 2 ms at 250 Hz, 1 ms at 500 Hz, and 0.5 ms at 1000 Hz.

The positive correlation between VTA vulnerability and the defibrillation success rate has been demonstrated previously[30–33]. VTA vulnerability was evaluated using the standard S1-S2 protocol [34], in which the S1s are the pacing stimuli and the S2 is the defibrillation shock with the four different frequencies. In a typical application of the S1-S2 protocol, the cycle length of the S1 pacing pulse train should be slightly shorter than the heart’s intrinsic basic cycle length. In our experiment, the intrinsic basic cycle lengths of the isolated rat hearts were 230–350 ms; thus, S1 cycle lengths of 200 ms were used in the study. S2 refers to one defibrillation shock immediately following the S1 pacing pulse train. The brief interval between S1 and S2 is called the S1-S2 coupling interval [34–36], which was set at three values in this experiment: 50, 100, and 150 ms. In each heart, the four defibrillation waveforms (125, 250, 500, and 1000 Hz) were successively used as the S2 in the vulnerability test, and each waveform was applied three times by using the respective S1-S2 coupling intervals (50, 100, and 150 ms). Therefore, each heart received fibrillation induction 12 times. If VTA was induced, then a defibrillation shock with the same frequency as the induction shock was applied when the VTA persisted for longer than 2000 ms, and the defibrillation success rate was calculated accordingly.

Six hearts were used in this study. In the first three hearts, the sequence of the S1-S2 coupling intervals was 150 ms, 100 ms, and 50 ms. In the other three hearts, such a sequence was reversed to become 50 ms, 100 ms, and 150 ms. This process was used to minimize the confounding effect of the order of the S1-S2 coupling intervals on VTA inducibility. A 3-min recovery period was added between consecutive shock applications to allow the tissue sufficient resting time.

### Data analysis

The heart responses to different shock frequencies were compared using a one-way analysis of variance (ANOVA) between the four groups and a two-sample *t* test for paired data between two of the four groups. All values were reported as means ± standard deviation. We set a shock frequency of 125 Hz for a control group and compared it with other shock frequencies. Statistical significance was represented by *p* < 0.05.

## Results

The experiment setting is illustrated in Fig 1, and the details of the experimental design are described in the Methods section.

### Tissue response to high-frequency shocks

**Fig 2:**
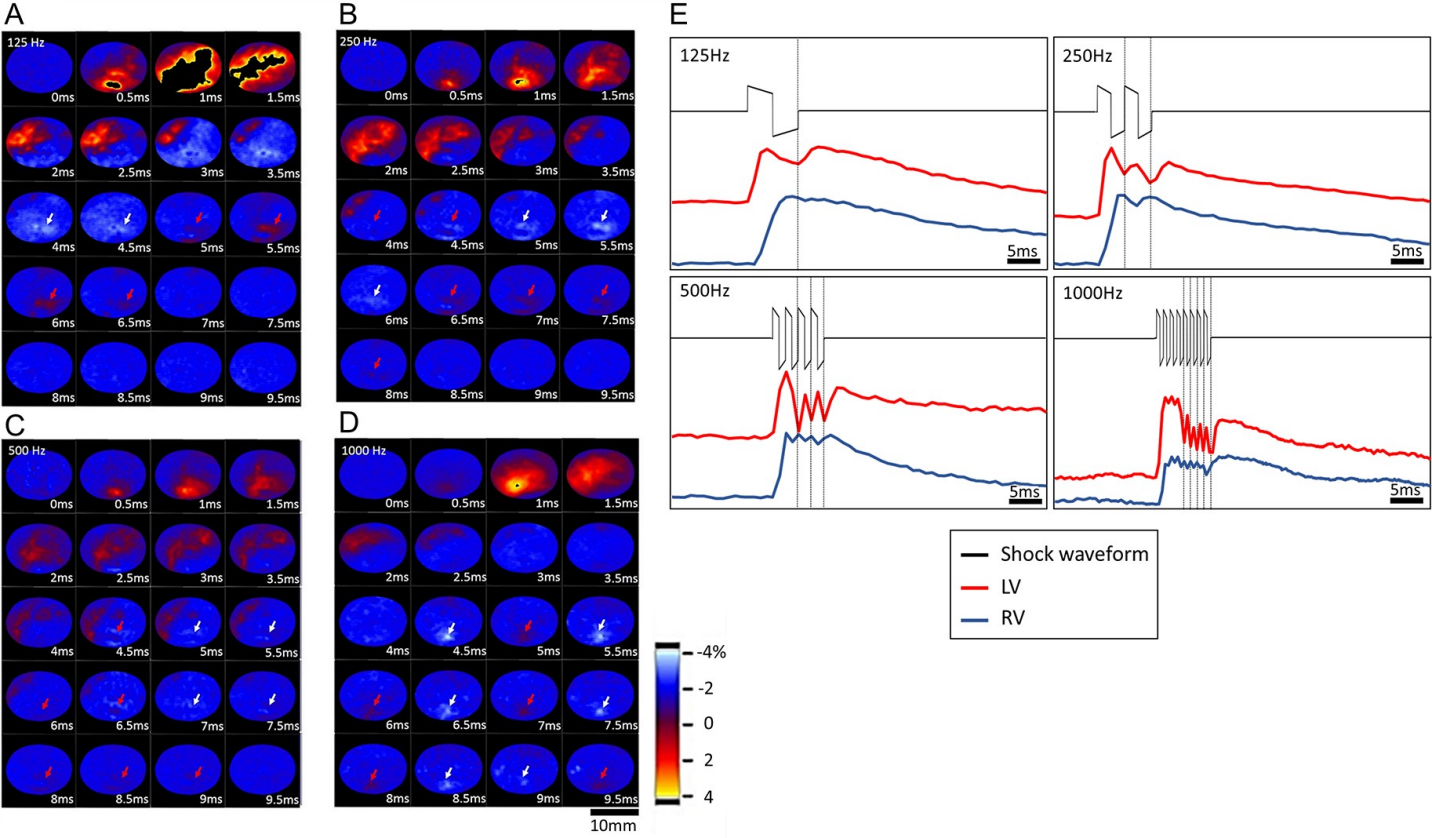
Different frequencies of defibrillation observed using optical mapping. (A–D) Whole-heart optical potential response to shocks of 125–1000 Hz observed from the difference in fluorescence intensity between successive frames, displaying wavefront polarization. Red arrows indicate depolarizing virtual cathodes; white arrows indicate repolarizing virtual anodes. These virtual electrode responses were observed in only four of the six rat hearts. (E) Comparison of shock waveforms and shock responses of the virtual electrodes on the left and right ventricles of the four shock frequencies.

Tissue response observed using optical mapping revealed that the virtual cathode and anode continued to appear backward and forward on the heart surface during electrical shocks. The high-frequency frames illustrated in Fig 2 were constructed using a “difference” mode of image processing in which each frame was obtained by subtracting the previous raw fluorescence image from the current one. The heart position is displayed in the inset of Fig 1A. The heart area was encircled by the region-of-interest tool. The area outside of the heart was masked in black. The tissue areas with depolarizing optical potential are shown in red and yellow, whereas repolarizing areas are in blue and white. A change in tissue polarization was observed when the colors in the frames changed from white to red or vice versa. The virtual electrode effect was observed in four of the six hearts (66%). For all the shock frequencies, postshock tissue responses demonstrated virtual electrode effects by displaying the recurrent virtual cathode (red spot) and anode (white spot) in opposite locations on the heart (Fig 2A–2D). The original file is included in S1 Appendix. These graphs indicate that virtual electrodes appeared most obviously in areas adjacent to the shock electrodes and were easier to observe in higher-frequency shocks. Optical potentials at the virtual electrode locations as indicated in Fig 2A–2D are plotted in Fig 2E. Each panel in Fig 2E displays the shock waveform (top trace) and the optical potential (lower traces). The optical potentials were measured near the physical electrodes attached to the left (middle red trace) and right (lower blue trace) ventricle. These optical potentials consistently exhibited virtual electrodes of opposite polarities following shocks of all four frequencies. The vertical lines mark the valley, or virtual cathodes, in the red traces obtained from the left ventricle. Corresponding to these valleys are the local peaks, or virtual anodes, in the blue traces.

**Fig 3:**
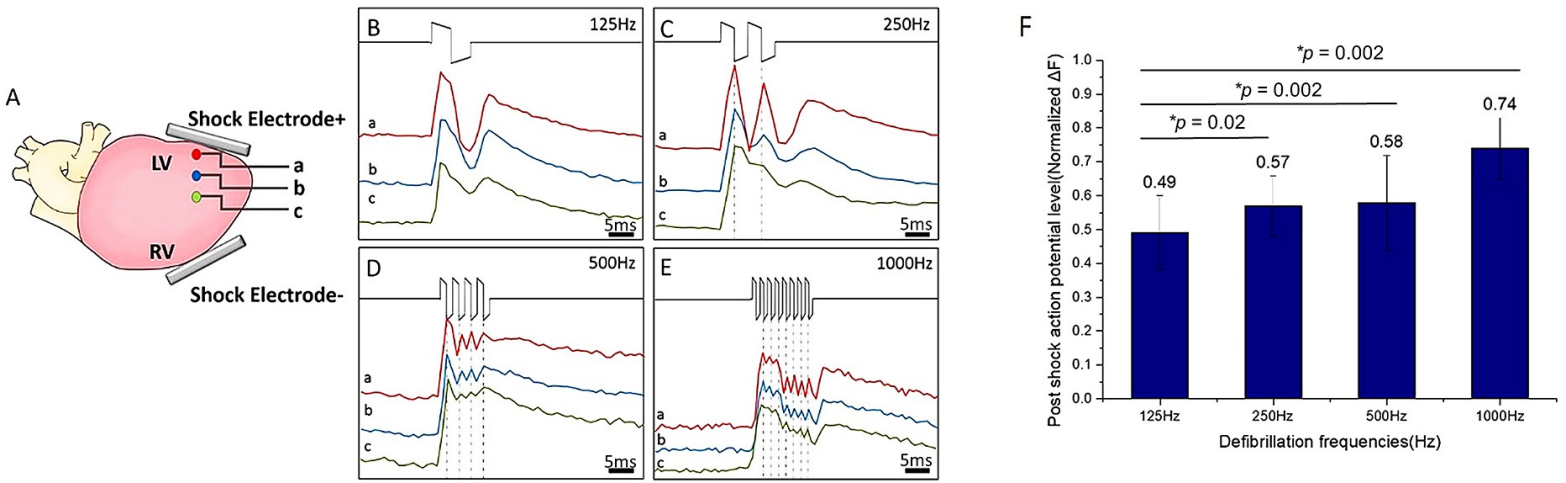
Different frequency responses at the midpoint of the heart and comparison of postshock action potential. (A) Three recording sites between the cathode and the midpoint on the heart. (B–E) The 125–1000-Hz shock-induced optical potentials. Vertical lines indicate instances of the shock waveform switching from positive to negative potential. The x-axis is the time scale (ms), and the y-axis is relative brightness with arbitrary units. (F) Postshock optical potential level at point (*a*) for the four shock frequencies (* represents *p* < 0.05).

To examine the tissue responses along the two shock electrodes, three points (a–c) were separately marked in red, blue, and green from the cathode to the midpoint between the two shock electrodes (Fig 3A). The length from *a* to *c* was approximately 0.5 mm. Thus, the distance between adjacent points was approximately 0.25 mm. The optical potentials at these points were extracted from the image sequence to evaluate the propagation of defibrillation responses from the electrode to the center of the heart. The optical potential resulted from the biphasic (125 Hz) and 250-, 500-, and 1000-Hz shocks along the shock electrode are displayed in Fig 3B–3E. In general, the optical potential amplitude of the shock response gradually decreased as the recording site moved away from the shock electrodes. The postshock optical potential levels, measured 8 ms after the shock, are illustrated in Fig 3F. The four frequencies of postshock potential levels were analyzed using one-way ANOVA (*p* = 0.02, α = 0.05) and were significantly different (*p* < 0.05). Among the four defibrillation waveforms, the 500-Hz (0.58 ± 0.15, normalized ΔF) and 1000-Hz (0.74 ± 0.09, normalized ΔF) shocks produced higher postshock optical potential than that of the standard 125-Hz biphasic shock (0.49 ± 0.11, *p* = 0.002). In addition, the 250-Hz (0.57 ± 0.09, normalized ΔF) shock was significantly different from the 125-Hz shock (*p* = 0.002). High postshock action potential could prolong the refractory period, thus increasing defibrillation efficacy.

### Tissue activation from shocks

**Fig 4:**
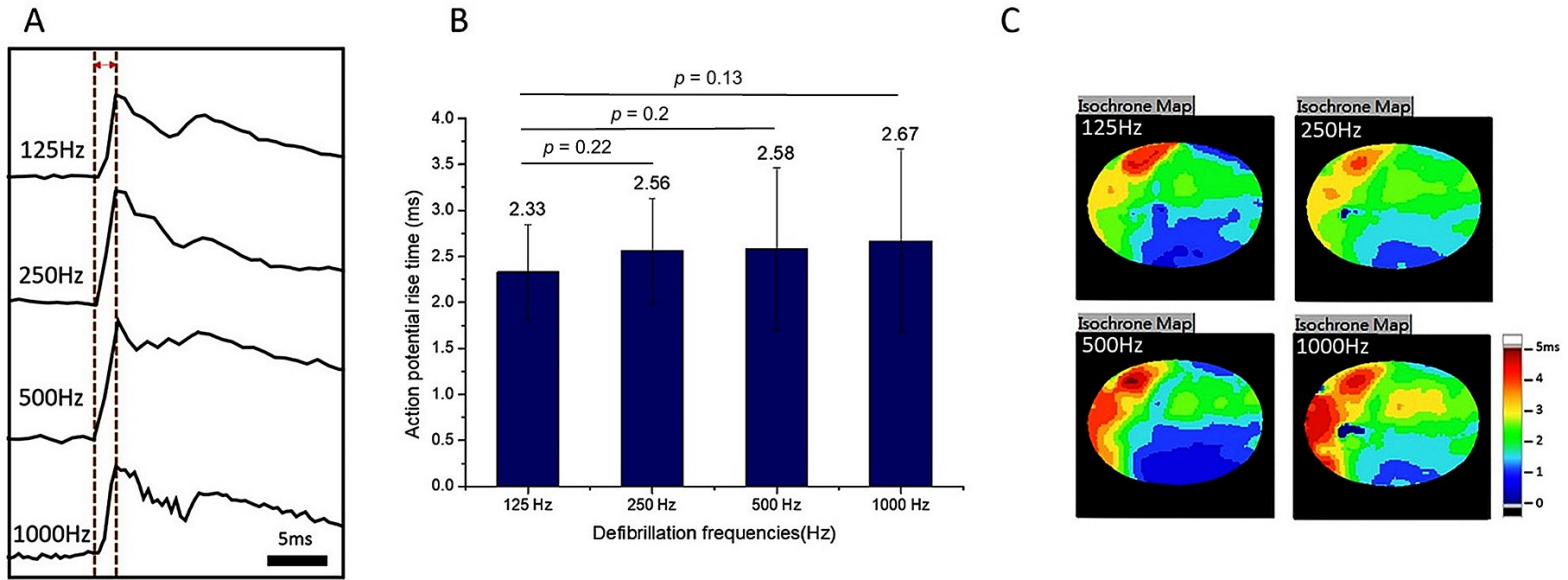
Comparison of action potential rise time and shock isochronal maps among the four shock frequencies. (A) Responses to the four shock frequencies at point *c* in Fig 3B–3E were combined for comparison. The x-axis is the time scale (ms), and the y-axis is the relative brightness with arbitrary units. (B) Comparison of the optical potential rise time resulting from the four shock frequencies. (C) Isochronal maps of the shock-induced tissue response in the entire heart.

“Optical potential rise time” is defined as the occurrence time from the rising point to the first peak of the optical potential shock response, as indicated by the red vertical lines in Fig 4A. Fig 4B displays a comparison of optical potential rise time. The rise time of the 50-ms S1-S2 interval was not included because an S1-S2 interval of 50 ms was too short, and shocks delivered during the eighth S1 action potential duration caused an incorrect estimate of the optical potential rise time. Thus, the figure presents only statistics of the rise time from the S1-S2 intervals of 100 and 150 ms. We calculated the average action potential rise time in the four frequencies. No significant differences in rise time were observed for 250 Hz (2.56 ± 0.57 ms, *p* = 0.22), 500 Hz (2.58 ± 0.88 ms, *p* = 0.20), or 1000 Hz (2.67 ± 1.00ms, *p* = 0.13) compared with 125 Hz (2.33 ± 0.52 ms).

Isochronal activation maps were created to compare the activation pattern of the four shock frequencies. Fig 4C presents optical mapping isochronal maps suggesting that shocks with the same total energy in four shock frequencies produced the same action potential propagation pattern and speed, which are consistent with the optical potential rise time profiles.

### VTA vulnerability

**Fig 5:**
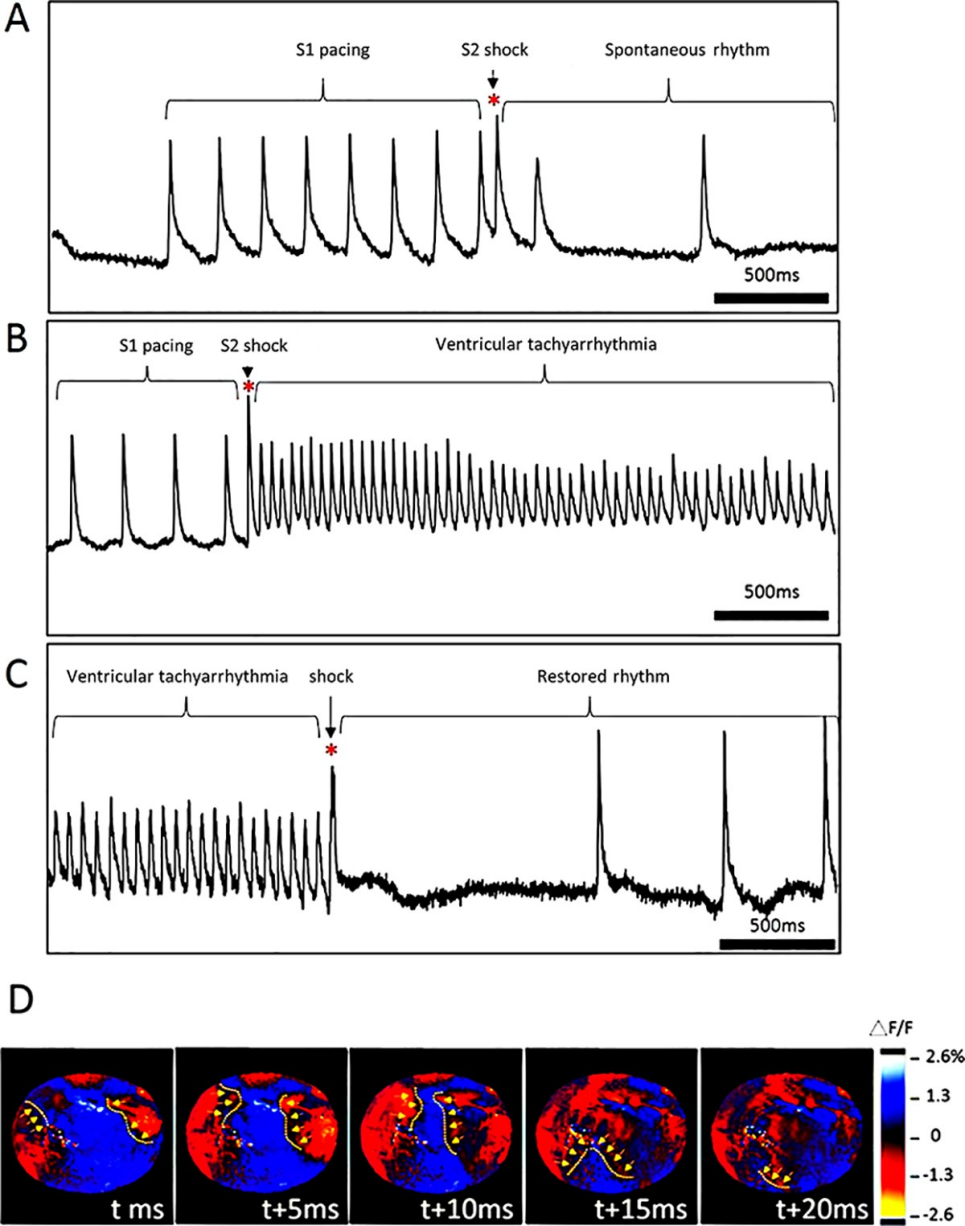
S1-S2 protocol-induced ventricular tachyarrhythmia (VTA) observed using electrocardiography and VTA observed using optical mapping. (A) Example of optical potential from S1-S2 pacing protocol without VTA induction. After applying eight S1 pacing pulses, S2 appeared as a premature stimulation (S1-S2 interval: 50 ms). (B) Successful VTA induction by a 1000-Hz shock (S1-S2 intervals: 100 ms). (C) Successful defibrillation by a 1000-Hz shock followed by the restored rhythm. (D) VTA wavefront observed using optical mapping. The yellow dotted lines and arrows indicate the direction of VTA wavefront propagation.

The standard S1-S2 stimulation protocol was used to evaluate the difference in ventricular vulnerability among different shock frequencies. Fig 5A presents an optical potential trace from the S1-S2 pacing protocol that failed to induce VTA. The shock was delivered after eight S1 pacing pulses but could not induce VTA. In addition, an example of successful VTA induction is presented in Fig 5B. In this example, the shock successfully induced fibrillation. After VTA induction, a shock was asynchronously delivered to terminate the fibrillation (Fig 5C). Optical mapping was performed during the induction and defibrillation procedures. An example of the VTA wavefront propagation is marked in optical mapping frames in Fig 5D.

**Fig 6:**
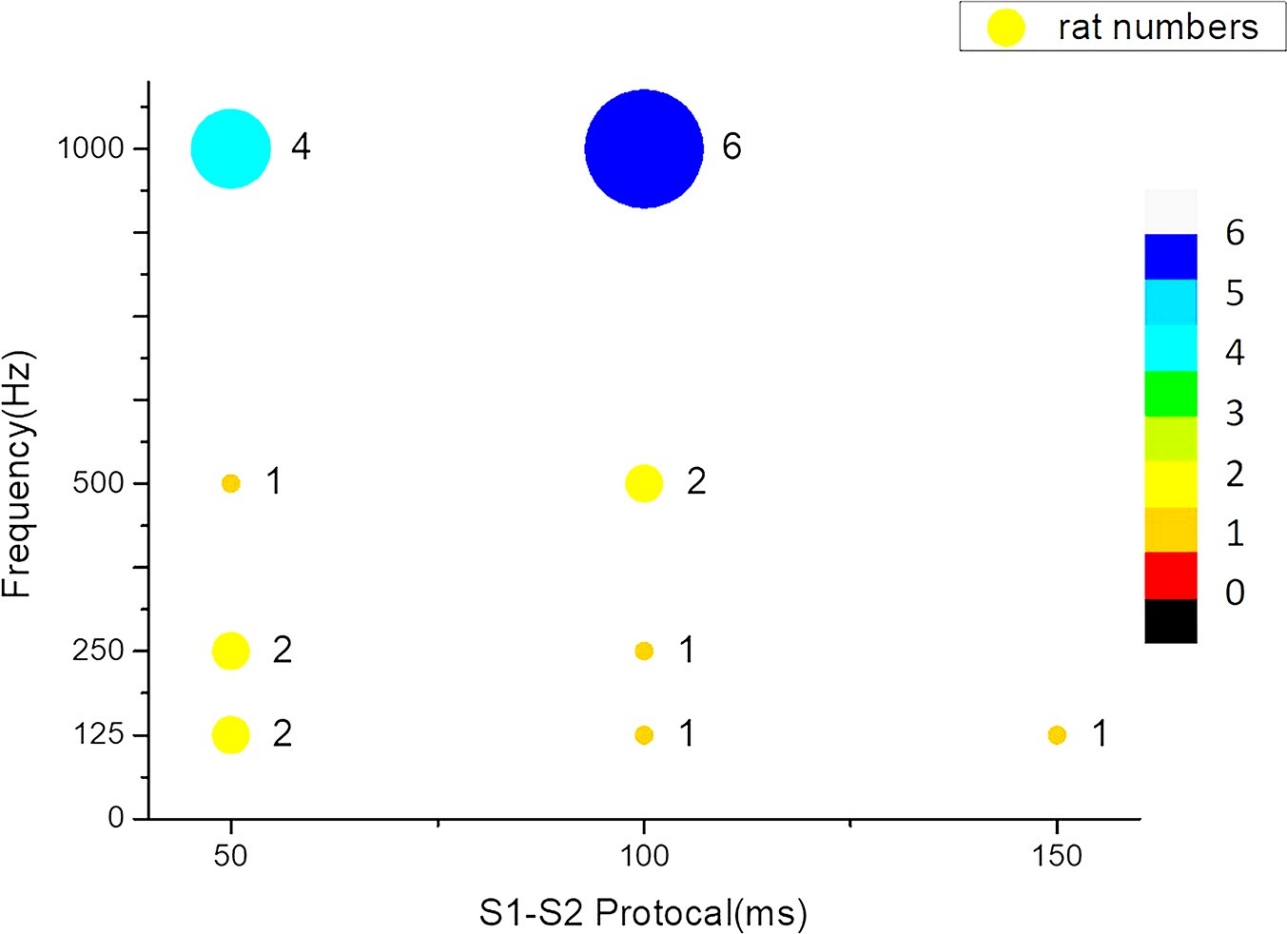
Ventricular tachyarrhythmia vulnerability bubble plot of S1-S2 coupling intervals and shock frequencies.

The upper limit of ventricular vulnerability is defined as the highest energy that can induce fibrillation [37]. However, the energy was constant during defibrillation with different frequencies in this research. Thus, VTA vulnerability is defined as the ratio of cumulative times of successful VTA induction over the number of S2 shocks delivered in the experiment. VTA induction was considered successful when the induced VTA persisted for more than 2000 ms [38]. VTA vulnerability increased as the shock waveform frequency increased (Fig 6). The 1000-Hz shock had the highest vulnerability among all the frequencies, and shorter S1-S2 intervals (50 and 100 ms) were correlated with higher vulnerability. The highest VTA vulnerability of the 100-ms S1-S2 interval was six times higher than that of the biphasic (125-Hz) shock. However, all the S1-S2 interval and frequency groups exhibited nonsignificant differences (*p* > 0.05) in the one-way ANOVA and *t* test. Regarding the defibrillation success rate measurement, after successfully inducing VTA, the subsequent defibrillation frequency was the same as the induction frequency. Thus, the defibrillation success rate was 50% (2 of the 4 hearts) for 125 Hz, 50% (2 of the 3 hearts) for 250 Hz, 33% (1 of the 3 hearts) for 500 Hz, and 70% (7 of the 10 hearts) for 1000 Hz.

## Discussion

The main findings of this study are as follows: (1) temporal sawtooth patterns can be observed using optical mapping during high-frequency shocks, (2) repeated virtual electrode responses prolonged postshock action potential and tissue refractoriness, (3) high-frequency defibrillation waveforms coupled with short S1-S2 intervals produced the highest VTA vulnerability and defibrillation success rates, and (4) the action potential rise time and isochronal maps of shock-induced optical potentials are the same irrespective of shock frequency.

Our results experimentally demonstrated for the first time that the entire heart can react to biphasic high-frequency shock waveforms of up to 1000 Hz. Shocks at even higher frequencies (>1000 Hz) were not evaluated in the current study because of the low signal-to-noise ratio in optical recording with high frame rates. Moreover, because the optical mapping camera frame rate was 2000 frames/s, according to the Nyquist sampling theorem, the highest shock frequency that could faithfully reveal the tissue response was 1000 Hz. Rapid polarity-reversing virtual electrodes and prolonged tissue refractoriness from high-frequency shocks are novel findings that could have crucial implications in defibrillation research. Because of the significant correlation between VTA vulnerability–related shock strength and defibrillation success [35], high-frequency defibrillating waveforms could achieve higher defibrillation success rates than those of conventional biphasic waveforms, and our study demonstrated this result.

High-frequency response has been discussed in many studies. Weirich et al. [39] applied alternating-current (AC) defibrillation waveforms with frequencies of 30–1000 Hz on guinea pig hearts and demonstrated that the fibrillation threshold increased when high frequencies were applied. One study demonstrated that 200 Hz had a higher defibrillation success rate than 1000 Hz did; however, the study had limited sampling frequencies [40]. High-frequency AC stimulation (1000–2000 Hz) can also inhibit cardiac excitation and generate reversible conduction blocks in cardiomyocytes [41]. In our study, virtual electrode responses and temporal sawtooth patterns were clearly observed when using the optical mapping technique. Nevertheless, our study was constrained by the optical mapping frame rate at 1000 Hz; thus, the virtual electrode reaction in each heart could not be observed during most of the 1000-Hz shocks (66%).

High-frequency ACs (HFACs) have been applied in real and simulated animal models [42, 43]. HFACs can produce conduction block and sustained refractoriness in cardiac tissue. HFACs were applied during spiral wave reentry in cardiomyocyte monolayers (HFAC: 50–1000 Hz) and ventricular arrhythmias in Langendorff-perfused guinea pig (HFAC field: 50–200 Hz) and rabbit hearts (HFAC field: 200 Hz). These results demonstrated that HFAC field stimulation could generate reversible conduction block and terminate reentrant arrhythmias. Our study employed capacitor-discharged rectangular biphasic waveforms rather than AC fields; however, our results of sustained refractoriness are consistent with those of relevant studies. Furthermore, we demonstrated the virtual electrode mechanism that may play a major role in establishing conduction block and sustained tissue refractoriness.

In this study, spatial sawtooth patterns were not identified for any of the shock frequencies. However, a temporal sawtooth pattern was clearly observed in the plateau region of the optical potential after initial tissue activation for all shock frequencies (Fig 2E). The amplitude of the temporal sawtooth decreased as the recording site moved away from the physical shock electrode. The prolonged refractoriness and temporal sawtooth pattern may facilitate the penetration of electrical energy into deeper layers of heart tissue, which is preferred in defibrillation. Further studies are required to investigate the implications of these temporal sawtooth patterns.

The virtual electrode was clearly observed during shock delivery in this study. Wikswo et al. [15] demonstrated that monophasic stimulation can generate electrical virtual electrodes of opposite polarity at the stimulation site. The current research also demonstrated that the virtual electrode can be observed during shocks by using the optical mapping technique. Our results are the first to demonstrate the synchronization of virtual electrodes with high-frequency multiphasic shocks of up to 1000 Hz. Such a peculiar spatial pattern led to prolonged tissue refractoriness and could have crucial implications in determining defibrillation outcomes.

Biphasic waveforms are superior to monophasic waveforms because of their improved ability in terminating VTA [29, 44, 45]. In addition, triphasic waveforms have a significantly higher probability of shock success than biphasic shocks [27], and quadriphasic waveforms exhibit further improvement at higher energy levels [46]. Consequently, we used only biphasic shocks for comparison with multiphasic shocks. These results led to the speculation that multiphasic waveforms could improve defibrillation efficacy as the number of waveform phases increases. Sweeney et al. [47] proposed that the heart acts as a low-pass filter for high-frequency monophasic pulses. The authors demonstrated that the defibrillation requirement was approximately twice as high when the pulse frequency exceeded 1000 Hz. Our experiment involved the use of biphasic shocks rather than monophasic shocks; thus, the capacitor effect may not be obvious. In fact, such an effect was not observed in high-resolution optical mapping.

Although tissue response to shocks can be directly visualized using the optical mapping technique, such a method is limited to only recording electric propagation on the heart surface. In the optical mapping results depicted in Fig 2, the virtual anode and cathode appear at the opposite sites of the true cathode and anode, respectively. The initiation and termination of VTA are determined by many structural and dynamic factors [48]. The defibrillation shock can cause major changes to the electrical dynamics. Increased defibrillating frequencies likely have a strong influence on the intracellular and extracellular electrical current flow that disrupts wave propagation with the virtual electrode formation mechanism and causes prolonged refractoriness through recurrent virtual electrodes. In our previous research.

In analyzing electrical energy delivered to the heart, the heart may be modeled as a parallel circuit consisting of a resistance and a capacitance [49, 50]. The resistance equals the series combination of the effective series resistance of the turn-on transistor, current-limiting resistance, electrode resistance, and the heart resistance. For multiple phasic reversals of the shock waveform, due to the charging effect of the heart capacitance, the switching between phases is not instantaneous. Therefore the total energy delivered to the heart was not exactly the same among shock waveforms with different frequencies. Our results showed that the switching time between phases was less than 0.1 ms. Therefore, the difference in the delivered energy may not be significantly different among different-frequency waveforms. Nevertheless, consistent with the charge-burping model of defibrillation [51], the higher frequency shocks generated more phasic reversals in tissue responses and could underlie the higher efficacy in the defibrillation outcomes than the lower frequency ones.

This study had several limitations. First, each heart received 12 defibrillation episodes. Despite the resting period between shocks, multiple shocks may have caused heart tissue damage. Second, our system used single-capacitor discharge at the output stage. Because of the low energy consumption of defibrillating a small heart, exponential decay of the waveform during the shock was not readily detectable. We tested waveforms with equal energy, but the vulnerability test results could depend on other factors, such as the average amplitude of the shock waveform. The effect of these different waveform features on ventricular vulnerability may require further investigation. In addition, the setting and the order of the S1-S2 coupling intervals may affect the defibrillation results. Also, the experimental protocol of using fixed S1-S2 coupling intervals cannot precisely determine the vulnerable window, as the subtle 1–2 ms could be critical in determining the VTA inducibility. Finally, in optical mapping, the absolute value of membrane potential cannot be established reliably. Therefore, the changes in membrane potential resulting from the application of high-frequency shock waveforms could only be represented qualitatively.

## Conclusions

In this study, we designed a defibrillator with adjustable frequency and demonstrated that multiphasic shocks with frequencies of up to 1000 Hz can induce temporal sawtooth patterns and synchronous virtual electrode responses. Ventricular tissue is more vulnerable to high-frequency defibrillation shocks than to low-frequency ones. High-frequency defibrillation shocks may be further investigated to enhance defibrillation success with high efficacy and low energy consumption and thereby reduce heart damage.

## Supporting information

S1 Appendix Original optical mapping file for verification (125 Hz, 250 Hz, 500 Hz, and 1000 Hz). These files were recorded using a high-speed camera (MiCAM).
(7Z) Click here for additional data file.
